# Posterior versus direct anterior approach in revision hip arthroplasty using Kerboull-type plate

**DOI:** 10.1051/sicotj/2019040

**Published:** 2020-01-14

**Authors:** Tomonori Baba, Yasuhiro Homma, Yuta Jinnai, Hiroki Tanabe, Sammy Banno, Taiji Watari, Kazuo Kaneko

**Affiliations:** Department of Orthopedic Surgery, Juntendo University School of Medicine 2-1-1 Hongo Bunkyo-ku Tokyo Japan

**Keywords:** Direct anterior approach, Posterior approach, Revision hip arthroplasty, Kerboull-type reinforcement device

## Abstract

*Introduction*: The purpose of this study was to investigate revision with a Kerboull-type plate through the posterior approach (PA) and direct anterior approach (DAA) and compare the clinical outcome.

*Subjects and methods*: Fifty-four patients (56 hip joints) underwent revision surgery in which acetabular reconstruction was performed concomitantly using the Kerboull-type plate and allogeneic bone. Revision surgery through DAA was performed in 21 hip joints and these were compared with 34 hip joints treated through PA. There was no significant difference in the patient demographics between the DAA and PA.

*Results:* There was no significant difference between the operative times in the DAA and PA groups (203.2 ± 43.5 and 211.7 ± 41.8 min). There was a significant difference between the intraoperative blood loss in the DAA and PA groups (503.9 ± 223.7 mL and 703.8 ± 329.6 mL, respectively, *p* < 0.05). There was no significant difference between the modified Harris Hip Score in the DAA and the PA groups. The loosening of the acetabular component was observed in four cases (11.8%) in the PA group. In the DAA and PA groups, the 5-year survival rates were 100 and 85.7%, respectively. Recurrent dislocation of the hip was observed in six cases (one case in the DAA group (4.8%) and five cases in the PA group (14.7%)).

*Conclusions*: It was verified that the difference in the surgical approach of acetabular reconstruction concomitantly using the Kerboull-type plate and allogeneic bone graft influenced the postoperative outcome.

## Introduction

When loosening of the acetabular component occurs after total hip arthroplasty, revision surgery of the acetabular side is necessary, but it is technically difficult compared with normal total hip arthroplasty. The following options are available for acetabular revision surgery: 1) surgery with an acetabular component alone, 2) placement of an acetabular component with bone graft, and 3) acetabular reconstruction concomitantly using bone graft and a reinforcement device followed by placement of an acetabular component [1–6]. We employ massive bone graft using allogeneic femoral head and a cross plate (Kerboull-type plate) as a reinforcement device because acquisition of recovery of bone stock and a favorable long-term survival rate by this reconstruction method has been demonstrated [5–8]. In addition, we consider that the combination of this reinforcement plate and sufficient bone graft disperse loads on the acetabulum and enable loading early after surgery even in the acetabulum with a huge bone defect [9].

On the other hand, revision surgery causes complications in many cases, for which countermeasures are important [10, 11]. Soft tissue damage due to surgical stress loaded several times not only promotes muscle weakness around the hip joint but also increases the risk of postoperative dislocation. Thus, we focused on an intermuscular approach, direct anterior approach (DAA). Since soft tissue can be conserved in DAA, recovery after surgery is fast; it is considered associated with a low dislocation rate and reduction of postoperative pain, and a favorable postoperative outcome can be expected [12, 13]. In addition, fluoroscopy can be easily used during surgery through DAA because the patient is placed in the supine position during surgery. Appropriate use of fluoroscopy during surgery not only prevents improper implant placement but also enables accurate evaluation of leg length discrepancy and offset, to which improvement of the postoperative outcome can be expected. Thus, we hypothesized that acetabular reconstruction through DAA decreases complications during and after surgery, acquiring a favorable clinical outcome. The objective of this study was to retrospectively investigate revision with a Kerboull-type plate through DAA and previously performed revision surgery through the posterior approach (PA) and compared the clinical outcome.

## Subjects and methods

### Patients

**Figure 1:**
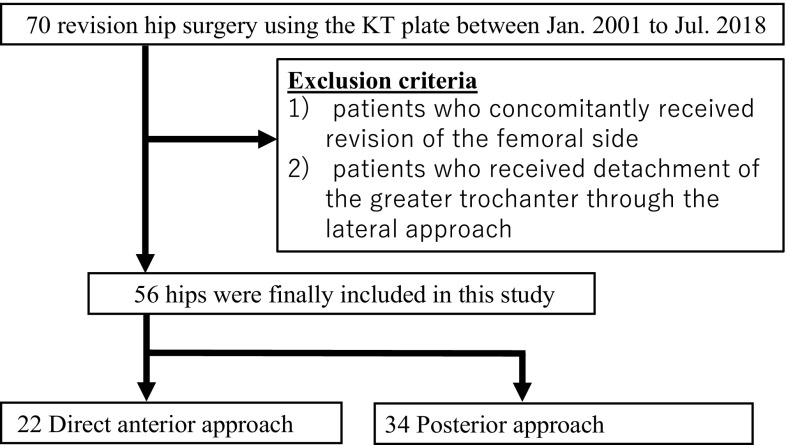
Flow chart of this retrospective study.

Sixty-eight patients (70 hip joints) underwent revision surgery in which acetabular reconstruction was performed concomitantly using a Kerboull-type plate (KT plate, Kyocera Medical, Osaka, Japan) and allogeneic bone of the femoral head between January 2001 and July 2018 (Figure 1). Excluding patients who concomitantly received revision of the femoral side and those who concomitantly received detachment of the greater trochanter through the lateral approach, the subjects were 54 patients (56 hip joints). After January 2012, revision surgery through DAA was performed in 22 hip joints (male: 2 hip, female: 20 hips) and these were compared with 34 hip joints treated through the PA (male: 2 hips, female: 32 hips). Table 1 shows the patient information. There was no significant difference in the age, height, and body weight between the DAA and PA groups. The cause of revision surgery was non-infectious loosening of the implant in all cases. The mean time to revision surgery was 17.0 years in the DAA group and 17.8 years in the PA group. The number of operations performed before this surgical procedure was 1.1 in the DAA group and 1.3 in the PA group. The Paprosky classification of the acetabular bone defect [14] was type 2a in 6 cases, 2b in 4, 2c in 7, 3a in 4, and 3b in 1 in the DAA group, and type 2a in 10, 2b in 7, 2c in 9, 3a in 6, and 3b in 2 in the PA group. The mean duration of follow-up was 3.8 years in the DAA group and 10.5 years in the PA group, showing a significant difference.

**Table 1:** Demographic characteristic of patients.

	DAA	PA	*P* value
Age (years)	71.7 ± 9.8	67.8 ± 8.9	0.1^b^
Sex (female, %)	90.9	88.2	0.9^a^
Height (cm)	148.7 ± 5.3	148.8 ± 6.2	0.9^b^
Weight (kg)	52.2 ± 7.6	51.4 ± 7.9	0.8^b^
Body mass index (kg/m^2^)	23.5 ± 3.6	23.2 ± 2.4	0.8^b^
Time to revision surgery (years)	17.0 ± 2.8	17.8 ± 2.4	0.8^b^
Primary disease			
Osteoarthritis	20 (90.9%)	34 (100%)	0.2^a^
Rheumatoid arthritis	1 (4.5%)		
Avascular necrosis of the femoral head	1 (4.5%)		
Paprosky classification			
2a	6 (27.3%)	10 (29.4%)	0.9^a^
2b	4 (18.2%)	9 (26.5%)	
2c	7 (31.8%)	9 (26.5%)	
3a	4 (18.2%)	4 (11.8%)	
3b	1 (4.5%)	2 (5.9%)	
Follow-up (year)	3.8 ± 1.9	10.1 ± 3.8	<0.01^b^

a Chi-squared test;

b Student’s *t-*test.

### Surgical technique

#### Surgical procedure via the DAA until reaching the hip joint

**Figure 2:**
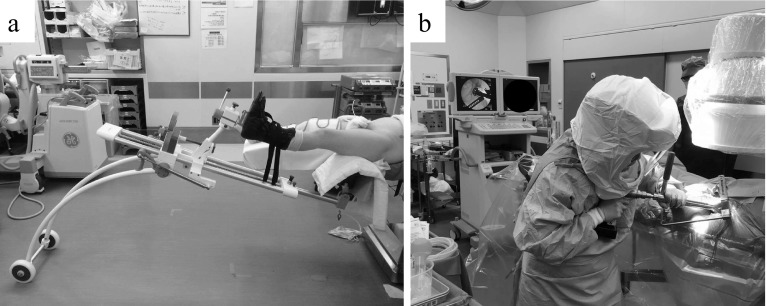
(a) Traction table (LECURE^®^); (b) Traction table is easy to use during intraoperative fluoroscopy.

Surgery was performed in the supine position using a traction table in all patients (Figure 2). Skin incision started from a position 2-fingerbreadth distal and 2-fingerbreadth lateral to the anterior superior iliac spine and an about 10-cm incision was made in parallel to a line drawn from the anterior superior iliac spine to the fibular head regardless of the skin incision made in the previous surgery. An incision slightly longer than the incision length in the normal first DAA-THA was made in consideration of the patient’s physique and the degree of implant migration. To prevent damaging the lateral femoral cutaneous nerve, the fascial incision site was set slightly lateral to the intermuscular region between the sartorius muscle and tensor fasciae latae muscle, and this intermuscular region was bluntly dissected. In cases in which the previous surgery was not performed through DAA, when a retractor was applied to the intermuscular region, the rectus femoris muscle and lateral femoral circumflex artery heading toward the hip joint could be confirmed on the medial side. This artery was ligated and cut beforehand. The capsular ligament anterior to the hip joint was incised in a Y-shape to subsequently re-suture as much as possible. Then, the capsular ligament was inverted to expose the sliding surface of the artificial hip joint. The inner head was disconnected in advance by traction of the hip joint. Operation of the acetabular side was performed after the stem neck was moved to a site posterior to the posterior acetabular wall by reducing traction of the hip joint and placing it in a slightly flexion position. The entire acetabular component became visible after moving the stem neck and the existing implant was removed using an appropriate method.

#### Surgical technique for acetabular reconstruction using the KT plate (Figure 3)

To place the hook of the KT plate on the tear drop, a space must be exposed. The tear drop position was in the lower deep layer of the surgical field if the approach were made via the lateral position, but in DAA it was rather easier to expose, being in the lower front. The transverse ligament was partially excised if there was not enough space for the hook. A template, the size of which was planned in advance, was inserted, and the size of the plate and the amount of bone defect were confirmed. Fluoroscopy was used as needed, and if the template was not sufficiently inserted, the osteophyte and acetabular floor region that interfered with the plate of the template were trimmed with a rongeur or a small acetabular reamer.

**Figure 3:**
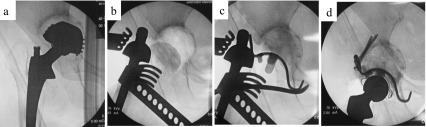
(a, b) For bone defects in the weight-bearing region of the hip joint, allogenic femoral head was trimmed, and temporary fixation with it as bulk bone was applied using Kirschner wire. (c) After confirming that the KT plate was placed at an appropriate position, the retractor was set on the hook, and the plate was firmly pressed and attached to the tear drop. (d) A polyethylene liner with an optimum size was fixed with cement.

When there was a bone defect in the weight-bearing region of the hip joint, the allogenic bone that had been trimmed after removing the cartilage was temporarily fixed with Kirschner wire as bulk bone. Any medial acetabular defects were filled with plate-shaped grafted bone. Since the KT plate has a dome shape, it was necessary to scrape the excess part of the temporarily fixed bone graft with a slightly smaller reamer; when the optimal size was reached, the real KT plate was inserted.

After confirming that the KT plate was in place, the retractor was applied to the hook and tightly pressed to the tear drop. The center of the KT plate was then struck and the gap between the bone graft and the host bone was crimped. The plate was temporarily fixed from the screw insertion part of the pallet with Kirschner wire, and then fixed permanently by inserting screws through the palette into the iliac bone. After fixing one screw, we confirmed the stability of the plate and the bone graft. Instability of either the plate or the bone graft indicated that either the position of the plate or the position or size of the grafted bone was incorrect; after determining the cause, the procedure was repeated. Once stability was confirmed, the final one or two screws were inserted. The remaining bone defect was filled with morselized bone as much as possible. A polyethylene liner of appropriate size was fixed by cement, to cover 40° of lateral opening angle and 20° of anterior opening angle. A trial femoral head was attached in order to evaluate the leg length and hip stability. Finally, the appropriate femoral head components were selected and the hip was reduced.

#### Surgical procedure via the PA until reaching the hip joint

Revision via the PA was performed using a standard operating table with the patient in the lateral decubitus position. The skin incision length was about 10 cm to 12 cm, centered on the greater trochanter. The gluteus maximus muscle was split in the direction of the fibers. If fibers remained, the short external rotators and the posterior capsule were incised. The acetabular components were removed after the hip joint was dislocated posteriorly. The surgical procedure for acetabular reconstruction with the KT plate was the same as for the DAA.

### Postoperative management

Postoperative treatment was standardized for all patients, including rehabilitation and pain management. Depending on the patient’s recovery and clinical condition, physical therapy was started on postoperative day one or two. All patients were permitted to perform full weight bearing as soon as possible, starting with a walker and aiming at independent walking. Irrespective of the approach used for revision, patients were instructed not to hyperflex, adduct, or internally rotate the lower limb. Patients were discharged once they could perform their daily activities safely.

### Assessment

All patients were followed-up by radiological and clinical evaluations at an outpatient clinic once every three months until one year after surgery, and every six months thereafter. The modified Harris Hip Score (mHHS) [15] was used to assess clinical outcomes. Survival analysis was performed using the Kaplan–Meier method, with the loosening of the acetabular component as an end point. Radiological looseness was defined as any of the following: 1) a screw or inferior hook was damaged or loosened; 2) the implant had moved 3 mm or more by horizontal or vertical migration, or the abduction angle of the acetabular component had changed by 3° or more; or 3) a radiolucent line of 3 mm or more was present in all three zones as defined by DeLee and Chanley [16].

### Statistical analysis

Continuous data were analyzed using Student’s *t*-test, and data grouped into categories were analyzed with the chi-squared test. A *p*-value < 0.05 was considered significant.

## Results

The intraoperative blood loss was significantly different between the two groups, but no significant difference was noted in the operative time (Table 2). There was a significant difference between periods before they were able to walk independently in the DAA and PA groups (6.6 ± 2.5 and 18.5 ± 7.1 days, respectively, *p* < 0.03). The mHHS was 53.7 ± 9.4 (DAA group: 52.8 ± 9.1; PA group: 54.2 ± 9.7) before the operation but significantly increased to 83.4 ± 12.4 (DAA group: 86.7 ± 10.3; PA group: 81.4 ± 13.2) at the time of the final evaluation (*p* < 0.01). No significant difference was observed between the DAA and the PA groups. The loosening of the acetabular component was observed in four cases (11.8%) in the PA group. In the DAA and PA groups, the 5-year survival rates were 100% and 85.7%, respectively (*p* = 0.16). Because there were no clinical symptoms in these cases, the course was observed without re-revision. Recurrent dislocation of the hip was observed in six cases (one case in the DAA group (4.8%) and five cases in the PA group (14.7%)).

**Table 2:** Surgical data and complications.

	DAA	PA	*P* value
Operative time (min)	203.2 ± 43.5	211.7 ± 41.8	0.6^b^
Intraoperative blood loss (mL)	503.9 ± 223.7	703.8 ± 329.6	<0.05^b^
Total complications	1 (4.5%)	9 (26.5%)	<0.05^a^
The loosening of the acetabular component	0	4 (11.8%)	
1. Inferior hook breakage	0	3 (8.8%)	
2. A change > 3 mm in implant position	0	1 (3%)	
Dislocation of hip	1 (4.5%)	5 (14.7%)	
Infection	0	0	

a Chi-squared test;

b Student’s *t-*test.

## Discussion

For revision hip arthroplasty, posterior and lateral approaches are widely used because the surgical visual field is favorable [5, 17–19]. The approach used by us in revision surgery using the Kerboull plate, the original of the Kerboull-type plate, has been reported by the developer of the plate, Marcel Kerboull et al., in which the lateral approach by detaching the greater trochanter was employed and acetabular reconstruction was performed concomitantly using the Kerboull plate and massive allogeneic bone graft. However, in patients with severe osteolysis, bone cortex of the greater trochanter was thinned and we often experienced false joint formation even though a hook plate was used for re-fixation after detachment of the greater trochanter. Thus, we adopted the PA not requiring detachment of the greater trochanter. Exposure of a favorable surgical field can be expected through the PA, but in the acetabulum, anterior exposure is slightly complex, while the posterior and upper exposures are superior [20, 21]. When anterior upper acetabular exposure is necessary, sufficient soft tissue dissection centering on the gluteus medius muscle is necessary. The placement position of the palette of the KT plate is slightly anterior and upper to the acetabulum and to insert a screw, dissection of more soft tissue is necessary. Moreover, it is not easy to secure a visual field of the obturator foramen, to which the inferior hook of the KT plate is applied, through the PA because it is present in the deepest layer. Furthermore, reconstruction of the short external rotators and posterior capsular ligament cannot be reliably carried out compared with that in primary THA due to the influence of previous surgery. Because of these reasons, high soft tissue invasiveness is inevitable for appropriate implant placement in surgery through the PA. Thus, we adopted DAA capable of conserving soft tissue in 2012. DAA is a true intermuscular approach capable of soft tissue conservation and it is low-invasive. In addition, it can be performed in the supine position in which fluoroscopy can be easily used. The effectiveness of fluoroscopy for accurate implant placement has been demonstrated, and we always use it when DAA is employed [22].

We previously reported the detailed surgical procedure of acetabular reconstruction with the Kerboull-type plate through DAA [23]. No serious complications, such as dislocation, infection, or periprosthetic femoral fracture, occurred in any patient after surgery. Tamaki et al. reported the short-term outcomes of 11 patients who underwent revision surgery using the Kerboull-type plate through DAA [24]. Their outcomes were mostly favorable, but fixation of the inferior hook of the Kerboull-type plate was fixed at an inappropriate position in a patient and the hook fractured 11 months after surgery, resulting in revision surgery. In our series, since the implant placement position was confirmed using fluoroscopy during surgery in all cases, the Kerboull-type plate was appropriately placed including the inferior hook and no fracture occurred in any case.

To our knowledge, no previous study has compared revision surgery between those through DAA and the PA performed in the same facility. In addition, the acetabular reconstruction method with the Kerboull-type plate and massive allogeneic bone was standardized in our study, being advantageous. Thus, the study could focus on only differences between the approaches for revision surgery. Many studies on primary THA demonstrated that recovery of muscle strength is faster, blood loss is smaller, and the dislocation rate is lower in surgery through DAA than through PA and soft tissue conservation is a factor of this. Blood loss was significantly smaller, recovery of walking ability was faster, and the dislocation rate was lower after revision surgery through DAA, similar to those in primary THA. In the only dislocation case in the DAA group, the first surgery was performed through the PA and anterior dislocation occurred. The existing femoral component in this case was an anatomical stem and anteversion was 60°. The cement cup was placed with a 20° anteversion angle not sufficiently considering strong anteversion of the stem. The patient was followed up with the use of a supporter capable of limiting both overextension and over external rotation of the hip joint for six months without re-operation. In the dislocation cases in the PA group, dislocation was posterior dislocation in all cases. The first surgery was performed through the PA in all cases, suggesting that surgery through the PA repeated several times induces cicatrization of posterior soft tissue and markedly reduces resistance to dislocation.

There are two limitations of the present study. Firstly, the number of cases was smaller in the DAA than PA group and the duration of follow-up was short. However, no previous study compared the two groups of revision surgery using the uniformed because the study was retrospective and patients with the use of cement and Kerboull-type plate; so we believe that this study is sufficiently valuable. Secondly, patients treated with revision surgery of both the acetabulum and femur were excluded. The reasons for the exclusion were that the implant used on the femoral side was not those with 2-stage surgery were mixed. Pure comparison of surgical stress, recovery of walking ability, and the dislocation rate may be possible by excluding these factors.

In conclusion, it was verified that the difference in the surgical approach of acetabular reconstruction concomitantly using a cross plate and allogeneic bone graft influenced the postoperative outcome.

## Conflict of interest

All authors have no conflict of interest to disclose.
